# Safety, feasibility, and quality of thulium laser en-bloc resection for treatment of non-muscle invasive bladder cancer

**DOI:** 10.1007/s11255-023-03752-5

**Published:** 2023-08-28

**Authors:** Ahmed Assem, Ayman Kassem, Mohamed Sherif, Amr Lotfi, Mohamed Abdelwahed

**Affiliations:** Faculty of Medicine, Kasr Alainy hospitals, Cairo University, Cairo, Egypt

**Keywords:** Bladder tumors, Thulium laser, En-block resection

## Abstract

**Background:**

Trans-Urethral Resection of Bladder Tumors (TURBT) is a critical step in diagnosis, staging and treatment of bladder tumors. Conventional TURBT (cTURBT) involves the electro-resection of the tumor into small fragments. This technique leads to concerns about the completeness of resection, under-staging, bleeding, cancer cell implantation, and most importantly, risk of tumour recurrence. To circumvent this, laser en-bloc resection of bladder tumors has been introduced.

**Objectives:**

Assessment of the safety, feasibility, and quality of Thulium Laser En-bloc Resection of Tumors (TmL-ERBT) for treatment of Non-Muscle Invasive Bladder Cancer (NMIBC) in various urinary bladder walls as a primary endpoint. The secondary endpoints were to investigate the feasibility of thulium laser use in the re-staging cystoscopy and to evaluate the learning curve of TmL-ERBT.

**Methods:**

This is a prospective observational study including all newly diagnosed patients, above 18 years old, with a urinary bladder mass ≤ 4 cm in maximal dimension (measured via bladder ultrasound or CT or MRI). All patients underwent TmL-ERBT under regional anaesthesia in a lithotomy position. All intraoperative complications such as obturator nerve reflex, bladder perforation, and significant bleeding were recorded. Postoperative variables such as the mean catheterization time, bladder irrigation fluid volume and duration, and the mean of hospital stay as well as the postoperative complications were recorded. All patients were risk stratified and managed according to EUA guidelines then followed by a surveillance regimen per 3 months for 6 months.

**Results:**

The study included 23 patients with a mean age of 53 ± 15.8 years. While 15 patients (65%) had a single tumor, the rest had multiple tumors, ranging from 2 to 3 in number with a total of 36 lesions. No cases required conversion to cTURBT and none of them experienced obturator nerve reflex or bladder perforation. Only one patient (4.3%) had an attack of clot urine retention. The mean hospitalization time was 31.2 ± 14.4 h and the mean catheterization time was 20.4 ± 13.3 h. The Detrusor muscle was present in 20 patients (87%) and the remaining 3 patients required a re-staging cystoscopy which was performed efficiently using thulium laser. None of the treated patients developed tumour recurrence during the follow-up period. In analysis, the duration of complete resection of 2–4 cm tumours was 23–27 min after the 7th case with a resection rate of 0.12–0.15 cm/min.

**Conclusion:**

TmL-ERBT is safe and feasible for complete resection of NMIBC with a short learning curve and adequate cancer control.

## Introduction

Non muscle invasive bladder cancer (NMIBC) represents about 80% of the primary bladder cancer. Transurethral resection of bladder tumour (TURBT) is the cornerstone of the diagnosis, staging and treatment of these tumors [1].

Complications of TURBT such as obturator nerve reflex, bladder perforation, post-operative bleeding, clot retention, removal of the tumors in piecemeal and inappropriate staging (absence of detrusor muscle or incomplete resection) [2] have paved the way for various procedures operating with different systems and application techniques for the treatment of NMIBC aiming at improving safety and efficacy of TURBT [2].

En-block Resection of Bladder Tumour (ERBT) is an alternative for piecemeal approach. Concurrently, many advantages have been attributed to ERBT such as lowering peri-operative complication rate, decreasing recurrence rate, and improving the resection quality. A newly envisaged goal is also to decrease the number of second TURBTs [3]. ERBT is most feasible in papillary tumors with distinct stalks, yet specimen extraction after en-bloc resection could represent a daunting challenge in case of large tumors. ERBT could be performed either by electrical-wire loop devices (monopolar and bipolar) or laser devices (holmium, thulium and CO2 diode laser).

Thulium laser was first introduced in urological practice in 2005 [4]. Thulium laser has been reported to provide smooth incisions; which could make the en-bloc resection more practicable and with a proper hemostasis [5]. Therefore, this study was designated to assess the safety, feasibility, and quality of thulium laser en-block resection of bladder tumour (TmL-ERBT) in various urinary bladder walls as a primary endpoint. The secondary endpoints were to investigate the feasibility of thulium laser use in the re-staging cystoscopy and to evaluate the learning curve of TmL-ERBT.

## Methods

This is a prospective observational study conducted between December 2021 and August 2022. The study was approved by the local committee of ethics (code: MS-93-2021).

A written consent was obtained from all participants after a clear and precise explanation of the nature, potential risks, and benefits of the procedure.

All treatment-naïve patients, above 18 years old, with a urinary bladder mass ≤ 4 cm in the maximal dimension (measured via bladder ultrasound or CT or MRI) were included. Whereas; patients having a tumour size ˃ 4 cm in the maximal dimension or with a history of previous TURBT or with a concomitant upper urinary tract tumour were excluded from the study.

All procedures were performed by one surgeon in attendance of a mentor who had 5 years of experience in laser resection of bladder tumours. Despite having no prior experience in the newer procedure, the primary surgeon was proficient in cTURBT with 10 years experience.

Under regional anaesthesia, unless contraindicated, TmL-ERBT was performed using 0.9% sodium chloride as an irrigation fluid while the patients were in a lithotomy position. A 550-μm optical laser fiber (RigiFibTM, LISA, Katlenburg, Germany) connected to a thulium laser device ((RevolixR, LISA, Katlenburg, Germany) was introduced in a working element of a 26F continuous-flow resectoscope (Karl Storz, GmbH, Tuttlingen, Germany) to start the tumouren-block resection after a formal urethro-cystoscopy to detect the location, size and number of masses. For en-block resection, energy of 1.5 J and frequency of 20 Hz were implemented, while the laser device was set to implement a power of 20 W in a pulsed wave manner for hemostatic purpose.

**Fig. 1:**
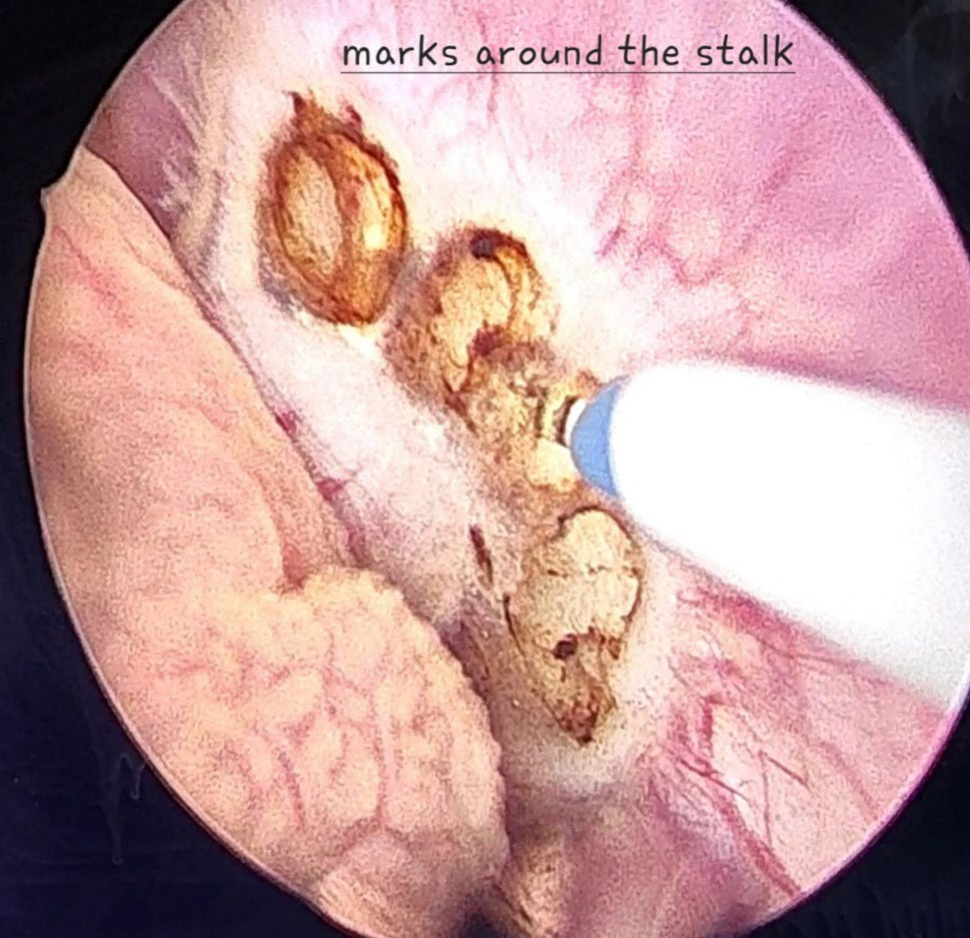
Demonstrates laser marks around the stalk of the bladder mass

**Fig. 2:**
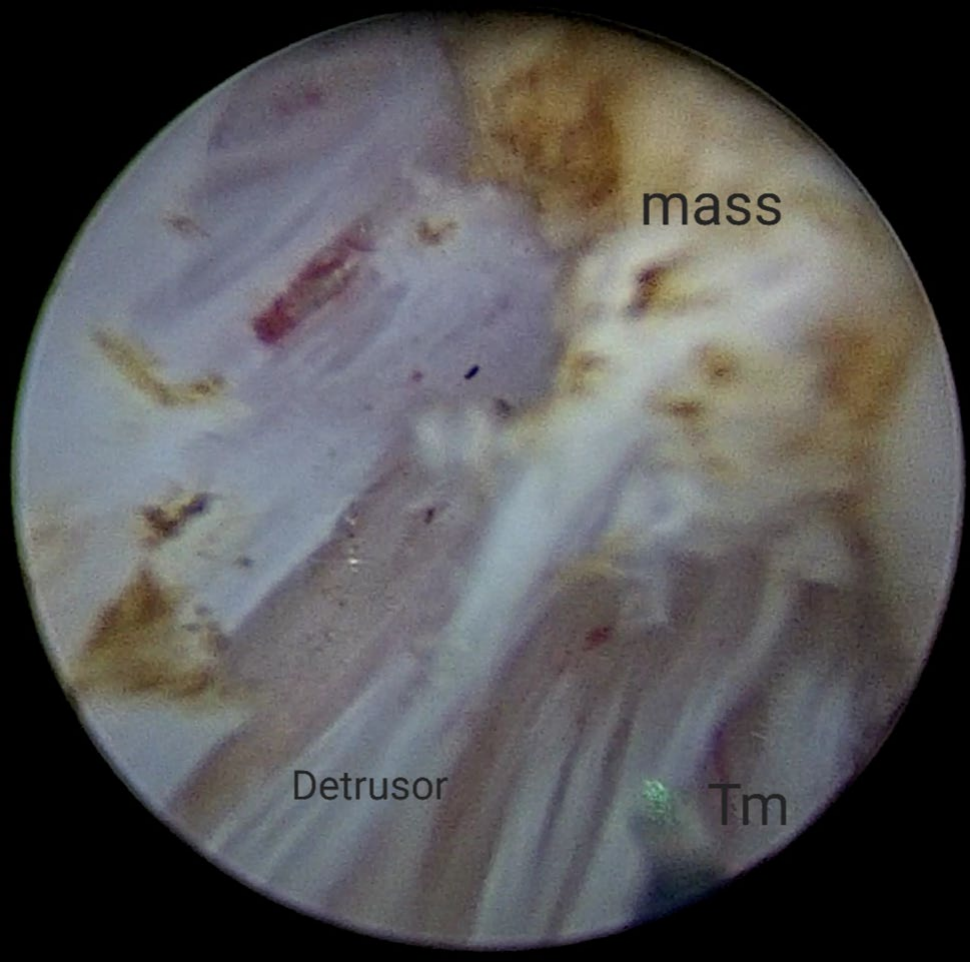
Illustrates the targeted plane (plane of cleavage)

**Fig. 3:**
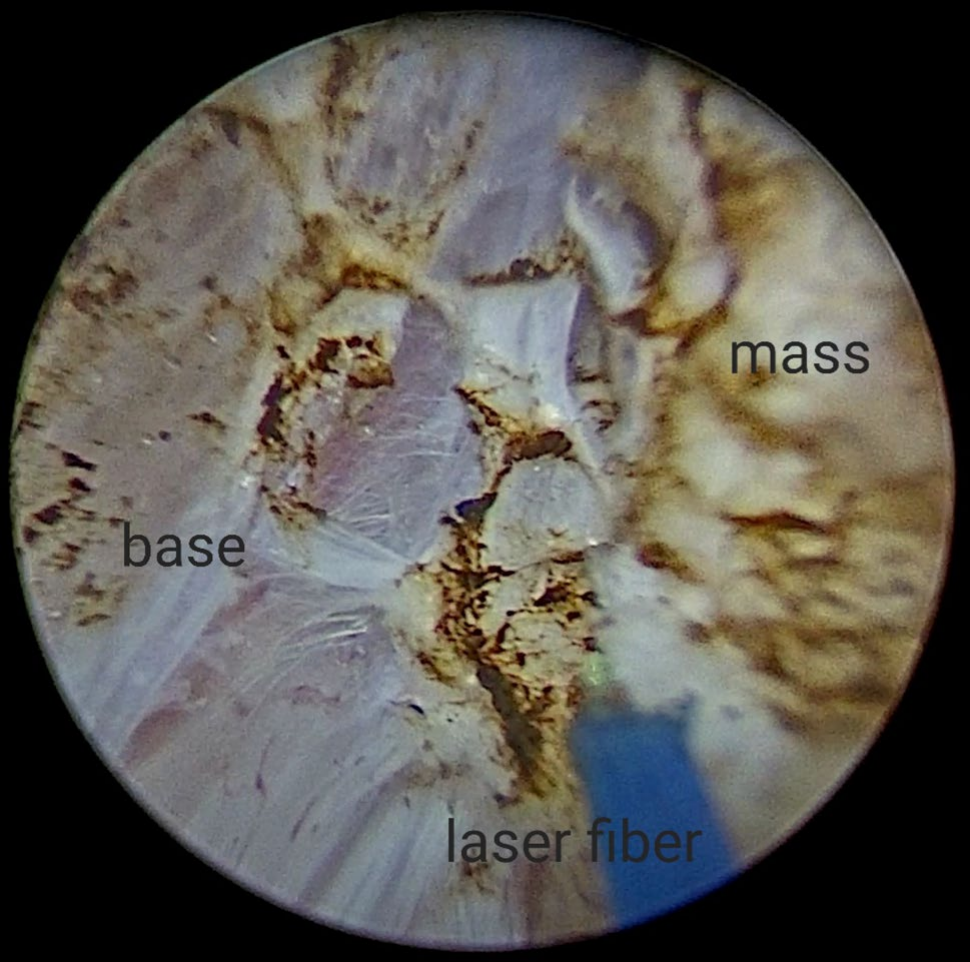
Demonstrates the enucleation of the mass with the underlying muscle base using laser resection along with mechanical dissection

For small villous tumours having distinct stalks, a 2–5 mm bladder mucosa incision was circumferentially marked around these stalks (Fig. 1) then deepened into the bladder wall until identification of the detrusor muscle fibres (the targeted plane or the plane of cleavage) (Fig. 2). Masses were then, facilely, uprooted. While; for non peduncleated masses, exertion of a slight pushing forward movement using the resectoscope tip (mechanical dissection) along with laser resection, after reaching to the targeted plane, was usually necessitated (Fig. 3).

For tumours located at the anterior wall of the bladder, with the assistance of a gentle and steady pressure on the suprapubic area, the tumour was enucleated in a similar manner to the above-mentioned technique while the resectoscope was rotated around. Whereas, for tumours located at the dome of the bladder, less mechanical dissection in a semi-filled bladder was exerted relying essentially on laser resection after reaching to the targeted plane to avoid the intra-peritoneal bladder perforation.

For re-staging purpose, after tattooing the previous resection site, the incision was deepened to the targeted plane without any mechanical dissection till the bladder biopsy was readily taken off.

In a way to prevent tumour cells spillage, as long as the tumour size was ˂ 2.5 cm, the mass was grasped, after enucleation, between the resectoscope cold loop and sheath then extracted with the resectoscope out of the urethra all together in one unit. While for tumours ≥ 2.5 cm, a cross sectional or longitudinal incision was always made to cut the tumour into two regular pieces while the tumour was still attached to its edge; incompletely resected (resembling a 'moving leaf attached to its branch'). These pieces were, then, removed out.

**Fig. 4:**
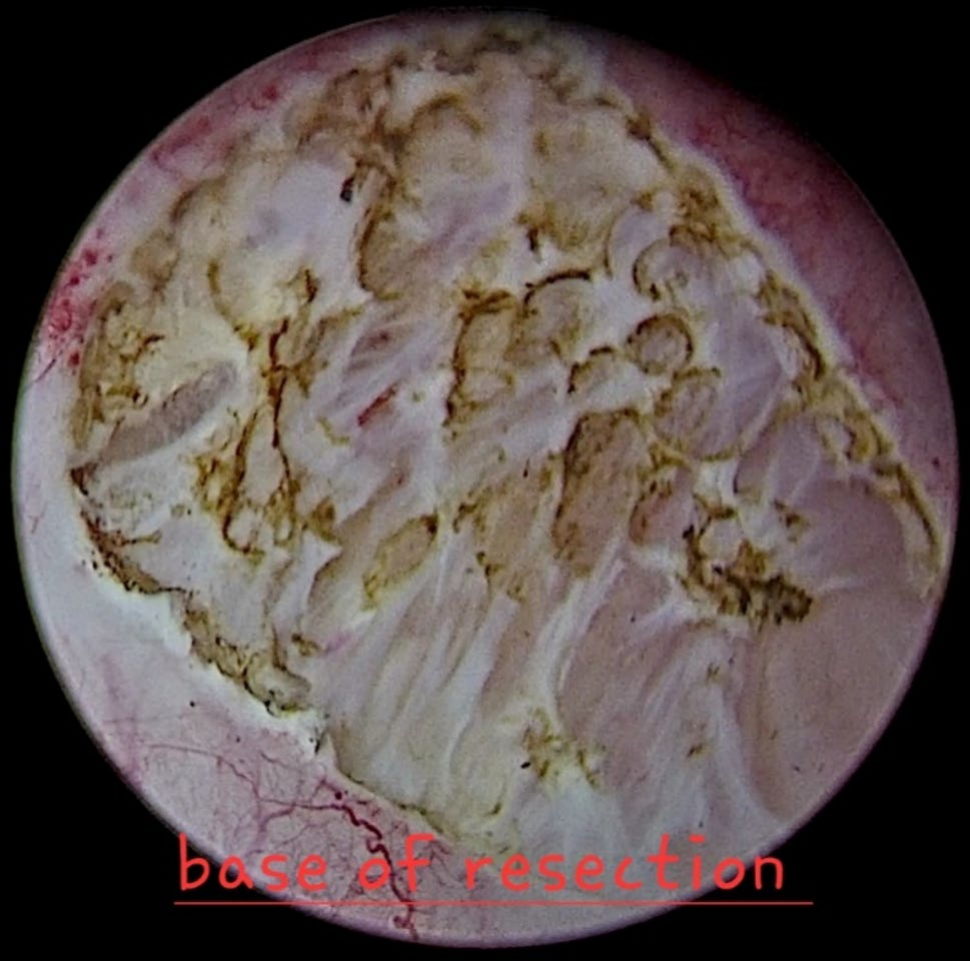
Illustrates the base of the bladder mass after TmERBT. Notably, these figures are archived photos which were taken during the study, compiled by Ahmed Assem, ahmed.assem@cu.edu.eg, with all rights preserved

By the end of resection, vaporization of the tumour base could be essential for asserting adequate hemostasis (Fig. 4) and continuous bladder irrigation (CBI), if required, was commenced after a 3-way catheter insertion. The specimens were prepared and sent for histopathological assessment.

All intraoperative complications such as obturator nerve reflex (ONR), bladder perforation, and significant bleeding were recorded. Preoperative and 2 h postoperative hemoglobin (Hb) and hematocrite (Hct) levels were measured and compared. In addition, the mean irrigation fluid volume and duration, mean catheterization time, and hospitalization time were estimated. All postoperative complications such as clot urine retention, intractable hematuria and their way of management were documented. The need for re-staging cystoscopy was also reported.

According to the histopathological assessment of the specimens, all patients were risk stratified and managed according to the EUA guidelines; including loading and maintenance doses of BCG intravesical instillation for intermediate and high risk groups, bladder ultrasound, urine cytology and regular surveillance cystoscopy per 3 months for 6 months.

The learning curve of TmL-ERBT was evaluated through comparing the duration of resection of 2–4 cm masses and detrusor muscle acquisition in the specimen at the beginning, the middle, and the end of the cohort.

### Statistical analysis

Using SPSS 22nd edition, categorical variables were presented in frequency and percentages, and were compared using Chi2 test. Quantitative variables were presented in mean and standard deviation and were compared using Mann–Whitney *U* test after normality testing. Sensitivity analysis was conducted to assess the predictive factors for adverse outcomes. Any *p*-value < 0.05 was considered significant.

## Results

The study included 23 patients with a mean age of 53 ± 15.8 years. Eighty seven percent of the patients were of male gender, while the rest (13%) were of female gender. Only one patient had a relevant family history of bladder cancer and 43.5% of the patients were active smokers.

During cystoscopy, a single mass was detected in 15 cases (65.2%), 2 masses were detected in 3 cases and 3 masses were detected in 5 cases; with a total of 36 masses in the whole cohort.

The most prevalent sites were the base of the bladder and right lateral wall followed by the dome of the bladder, left lateral wall, anterior wall, and the posterior wall (27.8%, 27.8%, 22.2%, 13.9%, 5.5% and 2.8%, respectively). The mean size of the first, second and third mass was 2.2 ± 0.7 cm, 1.6 ± 0.7 cm, and 1.8 ± 1.3 cm, respectively, and the mean time for resection was 22.9 ± 14.6, 16.1 ± 16.5 and 14.8 ± 19.8 min, respectively.

Intraoperatively, none of these patients experienced obturator nerve reflex or bladder perforation or conversion to cTURBT or required blood transfusion.

Postoperative CBI was required in 7 cases (30.4%) and only one patient developed an attack of clot urine retention for which CBI was needed for 9 h without the need for re-operation. The mean differences between preoperative and 2 h postoperative Hb and Hct were 0.7 ± 0.3 gm/dl and 3.9 ± 1.5% respectively, without significant statistical differences (*p*-value 0.75 and 0.385, respectively). The mean irrigation duration was 4.8 ± 4.2 h, and the mean irrigation volume was 4.8 ± 3.5 L, while the mean hospitalization stay was 31.2 ± 14.4 h and the mean catheterization time was 20.4 ± 13.3 h.

By histopathological examination of the specimens, 9 patients (39.1%) had stage Ta tumor, and 14 patients (60.9%) had stage T1 tumour. Twenty patients (87%) could be adequately assessed and staged in the first cystoscopy, whereas the detrusor muscle layer was not included in the specimen in 3 patients (13%); all were at the beginning of the study. Therefore, thulium laser re-staging cystoscopy was efficiently performed for these 3 cases and their stages were not, eventually, upgraded, i.e., T1 high grade tumours before and after the restaging procedure.

According to EAU risk categorization, 8 (34.8%) patients were of low risk, one patient (4.4%) was of Intermediate risk and 14 (60.8%) patients were of the high risk category. Loading and maintenance doses of intravesical BCG were instilled for intermediate and high risk groups. Grade 1 BCG toxicity was documented for three patients in the high risk group. All patients conformed to the surveillance regimen and none of them experienced tumour recurrence till the end of the follow-up.

In data analysis, the mass size and duration of resection could predict the postoperative hematuria requiring CBI. For mass size, using a cutoff point of 2.3 cm, with 85.7%, sensitivity and 81.2% specificity, the AUC was 88.4% and the p value was 0.004. For duration of resection, the cutoff point was 32.5 min, with 100% sensitivity, 87.5% specificity, the AUC was 97.3% and the *p*-value was 0.001. Moreover, the tumour location was not significantly correlated with the failure of detrusor muscle acquisition in the specimen (*p*-value 0.823).

**Fig. 5:**
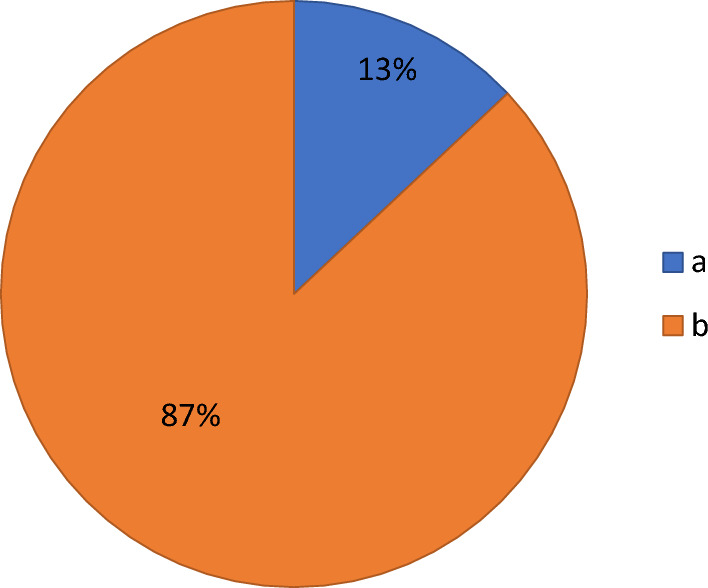
Represents the percentage of detrusor muscle detection in the specimen during the study; **a** the first 3 cases in the study needed re-staging procedure due to absence of detrusor muscle and **b** properly staged from the first TmERBT

**Fig. 6:**
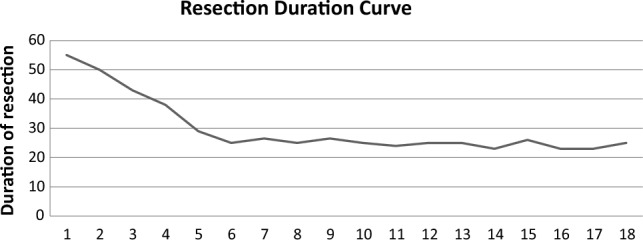
Demonstrates the resection duration for 2–4 cm tumours from the beginning till the end of the study (with a total of 18 patients)

Regarding the evaluation of the learning curve, inadequate staging due to the absence of the detrusor muscle in the specimen was only in the first three cases (Fig. 5) and the duration of complete resection of 2–4 cm tumours (18 cases) was 23–27 min after the 7th case with an enucleation efficiency of 0.12–0.15 cm/min, see Fig. 6.

## Discussion

TURBT is the mainstay of management of NMIBC. Conventional TURBT (cTURBT) entails the electro-resection of the tumor into small fragments; a way which leads to concerns about complete resection, cancer cell implantation, and consequently, increased risk of tumour recurrence [2]. Contemporarily, ERBT has been described to tackle this problem aiming at improving the safety and efficacy of TURBT [6].

The primary goal of ERBT is to provide adequate cancer control and better staging quality. This could be achieved by complete tumour resection and presence of detrusor muscle in the specimen, which may decrease the number of second TURBTs [7].

The second goal of ERBT is to lower the overall complications such as postoperative bleeding and bladder perforation. Postoperative bleeding is the most common complication after TURBT [8] and is usually reflected on the duration of bladder irrigation, catheterization as well as hospitalization times.

Bladder perforation is the most significant complication during TURBT and laparotomy, for intraperitoneal perforations, might be necessitated [1]. Aside from hindering the immediate postoperative intravesical chemotherapy instillation, tumour cells could be spilled out, following the bladder perforation, into the peritoneum or retroperitoneam resulting in unfavorable oncological outcomes [9].

The flow of electric current through the bladder wall into the obturator nerve, while resecting tumors at the lateral walls, brings about obturator nerve reflex (ONR). ONR occurs in 12–25% of patients undergoing bladder electrosurgery with a subsequent perforation rate of 2–10% [10, 11].

Many advantages have been reported on thulium laser use in ERBT. Owing to the continuous wave pattern and shallow penetration depth (0.2 mm) [12], thulium laser provides smooth incisions with a relatively clear vision [6]. Furthermore, the absence of the electric current in TmL-ERBT makes it safe with implantable cardiac pacing devices [13] and precludes the ONR, thereby decreasing the incidence of bladder perforation. Additionally, physiological saline is used as a perfusion fluid and therefore, reducing the occurrence of the TUR syndrome [13].

In the present study, TmL-ERBT was efficiently conducted for 23 patients with a total of 36 masses at various bladder walls. There was no a substantial statistical difference between the preoperative and postoperative Hb and Hct levels (p-value 0.75 and 0.385, respectively). The detrusor muscle was not detected in the specimen in the first 3 cases only (13%) and in terms of statistical significance; the tumour location had no impact on the detrusor muscle acquisition (*p*-value 0.823). None of these patients experienced ONR or bladder perforation and tumour recurrence was not documented during the 6 months follow-up period.

In consistence with the present study, Zhang et al. found that the detrusor muscle was not included in the specimen in 12.1% of TmL-ERBT arm group; which matches our study's results, albeit in a higher sample size (*n* = 149) [14]. On top of that, the detrusor muscle was, successfully, harvested in 100% of specimens during TmL-ERBT, as reported by some authors [6, 10, and 15], without a higher rate of bladder perforation.

Relatedly, Badway et al. have attempted to assess the feasibility of TmL-ERBT for tumours ˂ 4 cm in comparison to cTURBT and have found that TmL-ERBT is associated with a shorter operative time, a higher detrusor muscle detection rate, and a lower recurrence rate than cTURBT [16].

Many authors [5, 17, 18] pointed out that certain tumor locations might render some tumours, in approximately 30% of patients [3], inaccessible for ERBT “resection failure”. All tumours located at various bladder walls, however, were en-block resected in the present study without major intraoperative or postoperative complications. Therefore, the authors could agree with Migliari et al. [15] in the feasibility of TmERBT in all bladder walls.

The indications for restaging TURBT (re-TURBT) in NMIBC are still under debate [19], but it is essential for T1 high grade tumours with the lack of detrusor muscle in the specimen after the initial TURBT [20]. Regrettably, re-TURBT carries a higher rate of postoperative complications than TURBT [19]. Notwithstanding the limited number of re-staging procedures in the study, the authors claim that thulium laser restaging cystoscopy is a safe and easy to learn procedure whereby the detrusor muscle could be surely harvested after marking the previous resection site and detecting the targeted plane.

With acquisition of the detrusor muscle in the specimen after the first three cases “proper staging” and a constant resection rate after the first one third of the cohort (Fig. 5), 6–8 cases were enough to reach to a steady and efficient resection; denoting the short learning curve of TmL-ERBT.

According to what we know, this study is the first study to describe thulium laser use in the re-staging cystoscopy and also, to evaluate the learning curve of (TmL-ERBT); however, the study is limited by the small sample size. Therefore, randomized prospective comparative studies on larger scales are warranted to further assess the feasibility of the described techniques and their outcomes in a longer follow-up period.

## Conclusion

TmL-ERBT is a safe and effective procedure for NMIBC in various bladder walls, apropos staging, and re-staging purposes, with a short learning curve, adequate cancer control and minimal postoperative morbidity.

## Data Availability

All data are available upon request.

## Abbreviations

TURBT: Trans-urethral resection of bladder tumours
cTURBT: Conventional trans-urethral resection of bladder tumours
re-TURBT: Restaging TURBT
ERBT: En-block resection of bladder tumours
TmL-ERBT: Thulium laser en-block resection of bladder tumours
NMIBC: Non muscle invasive bladder cancer
ONR: Obturator nerve reflex
EUA: European urological association
CBI: Continuous bladder irrigation
AUC: Area under the curve
